# Lysophosphatidic Acid Activates Peroxisome Proliferator Activated Receptor-γ in CHO Cells That Over-Express Glycerol 3-Phosphate Acyltransferase-1

**DOI:** 10.1371/journal.pone.0018932

**Published:** 2011-04-20

**Authors:** Cliona M. Stapleton, Douglas G. Mashek, Shuli Wang, Cynthia A. Nagle, Gary W. Cline, Philippe Thuillier, Lisa M. Leesnitzer, Lei O. Li, Julie B. Stimmel, Gerald I. Shulman, Rosalind A. Coleman

**Affiliations:** 1 Department of Nutrition, University of North Carolina, Chapel Hill, North Carolina, United States of America; 2 Departments of Internal Medicine and Cellular & Molecular Physiology, Yale University School of Medicine, New Haven, Connecticut, United States of America; 3 Oregon Cancer Institute, Oregon Health Sciences University, Portland, Oregon, United States of America; 4 Department of Screening and Compound Profiling, GlaxoSmithKline Inc., Research Triangle Park, North Carolina, United States of America; Pennington Biomedical Research Center, United States of America

## Abstract

Lysophosphatidic acid (LPA) is an agonist for peroxisome proliferator activated receptor-γ (PPARγ). Although glycerol-3-phosphate acyltransferase-1 (GPAT1) esterifies glycerol-3-phosphate to form LPA, an intermediate in the *de novo* synthesis of glycerolipids, it has been assumed that LPA synthesized by this route does not have a signaling role. The availability of Chinese Hamster Ovary (CHO) cells that stably overexpress GPAT1, allowed us to analyze PPARγ activation in the presence of LPA produced as an intracellular intermediate. LPA levels in CHO-GPAT1 cells were 6-fold higher than in wild-type CHO cells, and the mRNA abundance of CD36, a PPARγ target, was 2-fold higher. Transactivation assays showed that PPARγ activity was higher in the cells that overexpressed GPAT1. PPARγ activity was enhanced further in CHO-GPAT1 cells treated with the PPARγ ligand troglitazone. Extracellular LPA, phosphatidic acid (PA) or a membrane-permeable diacylglycerol had no effect, showing that PPARγ had been activated by LPA generated intracellularly. Transient transfection of a vector expressing 1-acylglycerol-3-phosphate acyltransferase-2, which converts endogenous LPA to PA, markedly reduced PPARγ activity, as did over-expressing diacylglycerol kinase, which converts DAG to PA, indicating that PA could be a potent inhibitor of PPARγ. These data suggest that LPA synthesized via the glycerol-3-phosphate pathway can activate PPARγ and that intermediates of *de novo* glycerolipid synthesis regulate gene expression.

## Introduction

Peroxisome proliferator activated receptor-γ (PPARγ) is a nuclear receptor that is highly expressed when pre-adipocytes differentiate into adipocytes [1]. PPARγ regulates gene expression by forming a heterodimer with the retinoid × receptor (RXR) and binding to PPAR response elements (PPRE) in the promoter region of target genes. Genes that are regulated by PPARγ are primarily involved in adipocyte differentiation and fatty acid metabolism [1]. Ligands for PPARγ include polyunsaturated fatty acids [2] and synthetically derived thiazolidinedione (TZD) ligands for PPARγ that improve insulin sensitivity in patients with type 2 diabetes [1].

**Figure 1:**
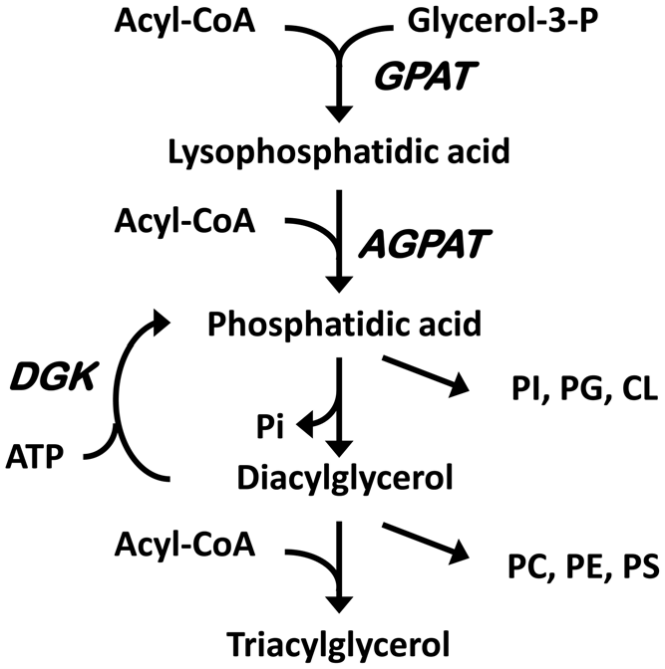
Pathway of glycerolipid synthesis. AGPAT, acyl-glycerol-3-phosphate acyltransferase; CL, cardiolipin; DGK, diacylglycerol kinase; GPAT, glycerol-3-phosphate acyltransferase; PC, phosphatidylcholine; PE, phosphatidylethanolamine; PG, phosphatidylglycerol; PI, phosphatidylinositol; PS, phosphatidylserine.

Lysophosphatidic acid (LPA) derived from hydrolysis of plasma membrane phospholipids is well established as a ligand for specific G-coupled cell-surface LPA receptors that generate an intracellular signal cascade that enhances cell proliferation [3]. Studies have suggested that exogenous LPA might also enter cells to activate PPARγ because LPA can bind to and displace rosiglitazone from PPARγ. Further, when LPA enters RAW264.7 monocytes, it activates a PPRE reporter driven by a PPARγ expression vector [4]. Although the naturally occurring ether analog of LPA, 1-O-octadecenyl-2-hydroxy-*sn*-glycero-3-phosphate (AGP), can enter cells and is a ligand for purified PPARγ (Kd ∼60 nM), the entry of LPA is more problematic [5]. Studies showing that LPA can activate the intracellular PPARγ reporter required dimethyl sulfoxide (DMSO) and tridentate sulfonamid to enhance the transport of exogenously added LPA across the cell membrane [4], [6]. Thus, although LPA can clearly activate PPARγ [7], it is not certain that exogenous LPA would normally function as a major physiological activator of PPARγ. Most LPA produced within cells is synthesized as an intermediate in the pathway of triacylglycerol and phospholipid biosynthesis, but it has been generally assumed that LPA formed by *de novo* synthesis from glycerol-3-phosphate (**Fig. 1**) has a role in lipid synthesis but not in signaling [8].

LPA is the direct product of glycerol-3-phosphate acyltransferase (GPAT), the rate-limiting step in glycerolipid synthesis [9] (**Fig. 1**). Because the absence of the mitochondrial isoform GPAT1 from liver causes a 50% decrease in LPA content [10], whereas adenovirus-mediated overexpression of GPAT1 increases LPA content 4-fold [11], we used a CHO cell line that stably overexpresses GPAT1 [12] to determine whether endogenously synthesized LPA could act as a PPARγ agonist. CHO-GPAT1 cells have a 3.8-fold increase in GPAT1 activity, contain a 2.7-fold higher triacylglycerol mass than control cells, and incorporate 3.4-fold more [^14^C]oleate into triacylglycerol, suggesting that the intermediates of glycerolipid synthesis might be higher than normal and that LPA levels might be elevated. We now provide evidence that LPA synthesized intracellularly via the pathway of triacylglycerol and phospholipid biosynthesis can activate PPARγ and that PA may inhibit PPARγ activity.

## Methods

### Cell Culture

Chinese hamster ovary cells (CHO) (CHO-K1, #CCL-61, ATTC) were maintained in MEM with 10% heat-inactivated fetal bovine serum, 100 units/mL penicillin and 100 µg/mL streptomycin (normal medium) at 37°C, 5% CO_2_. CHO cells that stably overexpress GPAT1 (CHO-GPAT1 cells) [12] were maintained in the same medium with 600 µg/mL G418 until studied. Expression of GPAT1 in CHO-GPAT1 cells is controlled by the pTet-Off plasmid (CLONTECH), so GPAT1 expression is repressed when doxycycline is present.

### Plasmids

pSG5 expression vectors encoding hPPARγ and mRXRα [13], [14] and the PPRE reporter plasmid ACOX2-tk-CAT [13], [15] were described previously. The pShuttle2 expression vector encoding hAGPAT2 was a generous gift from Dr. A. K. Agarwal (University of Texas Southwestern Medical Center) [16]. pcDNA3-DGKα WT and pcDNA3-DGKα Δ196, encoding constitutively active DGKα, were generous gifts from Dr. J. P. Walsh (Indiana University School of Medicine) [17]. The internal control reporter construct pRL-SV40 was from Promega. pcDNA3.1 was from Invitrogen.

### Transactivation Assay

CHO cells and CHO-GPAT1 cells were plated at 4×10^5^ cells/well in 6-well dishes. After 24 h, cells were transfected in Opti-MEM (GIBCO) with equal concentrations (0.4 µg) of expression vectors (PPARγ and RXR) and a PPRE-CAT reporter vector using FuGENE 6 (Roche). Transfections of PPARγ were in medium containing delipidated serum (HyClone). To monitor transfection efficiency, 0.1 µg pRL-SV40 was transfected as an internal luciferase control. Equal concentrations of DNA were transfected into cells at all times. As a control, cells that were not transfected with either the AGPAT2 expression vector or the vector expressing constitutively active DGKα were transfected with equal amounts of a negative control, i.e., pcDNA3.1 or pcDNA3-DGKα WT, respectively. Twenty-four hours after transfection, cells were treated with troglitazone (Cayman Chemical Company) or dimethyl sulfoxide (vehicle control). After further incubation for 24 h, cells were assayed for Renilla luciferase (LUC) (Renilla Luciferase Assay System; Promega). Chloramphenicol acetyltransferase (CAT) activity, a measure of PPRE activity, was determined using the CAT enzyme-linked immunosorbent assay kit (Roche). CAT reporter activity relative to LUC activity was calculated and plotted. Experiments were performed in triplicate, and for the statistical analysis, either Student's t-test, assuming equal variances, or ANOVA followed by multiple comparison of means with Bonferroni's multiple comparison test was used (GraphPad Prism, version 4 statistical software; GraphPad Software). Representative figures from each experiment are shown. Variation of luciferase sensitivity between experiments was responsible for the difference in the scale of relative CAT activity.

### Preparation of Fatty Acid:BSA Complex

Fatty acids were prepared in 20 mM stock solutions. Bovine serum albumin (BSA) (12.5%; essentially fatty acid-free) was prepared in MEM containing antibiotics and delipidated serum. Just before cell treatment, fatty acids were bound to BSA at a final concentration of 0.5 mM fatty acid and 1% BSA [18]. Cells were treated with a final concentration of 250 µM fatty acid.

### Preparation of Extracellular LPA and PA

1-Oleoyl-2-hydroxy-*sn*-glycero-3-phosphate (18∶1 LPA) and 1,2-dioleoyl-*sn*-glycero-3-phosphate (18∶1 PA) (Avanti Polar Lipids, Inc.) were dried under nitrogen and dissolved in 0.1% BSA immediately before use. Dioctanoyl glycerol (diC8∶0) (Sigma) was dissolved in dimethyl sulfoxide before use.

### Mass Spectrophotometry

CHO and CHO-GPAT1 cells grown to confluence in 150 mm dishes, washed with ice cold PBS, and scraped into 1 mL ice cold PBS. Cells were counted and stored at −80°C, and acyl-CoAs were extracted [19], [20]. To extract PA and LPA, cells were sonicated and homogenized in 1 mL of phosphate buffer (40 mM Na_2_HPO_4_, 30 mM citric acid, pH 4.0) plus an internal standard (heptadecanoyl-LPA). Two milliliters of butanol were added, and cells were rehomogenized and centrifuged at 4000 rpm for 15 min. Supernatants containing the LPA and PA were dried, redissolved in 1 mL water and applied to an Oasis HLB column. The column was washed once with water, and then LPA and PA were eluted with methanol. Authentic standards (acyl-CoA, TAG: Sigma Chemical Co. and 1,2-dodecanoin: IndoFine Chemical Co.) were used for quantification. Results are expressed as nanomoles analyte per gram protein extract. Analysis was performed with a bench-top tandem mass spectrometer API3000 (Perkin-Elmer Sciex) interfaced with TurboIonspray ionization source. The mobile phase was methanol and H_2_O with an isocratic gradient (50/50). Acyl-CoAs were ionized in negative ionspray mode [20]. Doubly charged ions and corresponding product ions were chosen as transition pairs for each acyl-CoA species for selective reaction monitoring quantitation. LPA and PA were ionized in TurboIonspray mode with MRM quantification of the parent ion (minus phosphate) and corresponding product ions of the fatty acid moiety.

### PPARγ LBD LEADseeker Scintillation Proximity Assay

Storage buffer [50 mM Tris pH 8, 50 mM KCl (100 mL)] was added to 500 mg of streptavidin-coated LEADseeker SPA beads (Amersham). After mixing for 30 min on a Nutator platform, the beads were centrifuged at 1500 rpm.for 10 min at 22°C. The bead pellet was resuspended in 100 mL of storage buffer at 5 mg/mL. At room temperature, the bead stock (5 mL) was diluted with 7.5 mL of assay buffer (50 mM Tris pH 8, 50 mM KCl, 2 mM EDTA, 5 mM CHAPS, 0.1 mg/mL BSA, 10 mM DTT) and 120–150 nM of biotinylated PPARγ LBD [21] was added to a final volume of 12.5 mL. The slurry was incubated for at least one hour with gentle agitation and then centrifuged at 1500 rpm for 10 min at 22°C. The bead pellet was gently washed with 12–15 mL of assay buffer, centrifuged as described previously, resuspended (45 mL assay buffer, 5 mL 1 mM biotin), and incubated for 1.5 h with gentle agitation.

Radioligand ([^3^H]rosiglitazone) stock was diluted to 150 nM in assay buffer, mixed with an equal volume of receptor-coated beads, incubated for 5 min, and added (10 µL) to 384-well assay plates (NUNC, 264675) containing 0.1 µL of compound. Final assay concentrations were 75 nM radioligand and 0.25 mg/mL PPARγ-LBD-coated beads. Fatty acids were diluted in ethanol: water (1∶1) with a starting concentration of µM and assayed as dose response curves. After a 2–15 h incubation at RT, covered and in the dark, the signal was determined at 613 nm using a Viewlux plate imager (Perkin Elmer). Non-specific binding was determined with 20 µM unlabeled rosiglitazone.

### Total RNA Extraction and Quantitative RT-PCR

CHO and CHO-GPAT1 cells were grown to 90% confluence, and RNA was extracted (RNeasy Midi Kit, Qiagen). Forward and reverse primers for mouse GPAT1 were 5′-ATGGACGCAAAGACATTCTGT-3′ and 5′-AAGATCTCCAGGAACTGCTG-3′, respectively. The corresponding FAM probe was 5′-CGTTGCTCCATGGGCATGTAGTTG-3′. The 18s primer and probe sequences were homologous for mouse, rat, and human (Forward: 5′-AGAAACGGCTACCACATCCA-3′; reverse: 5′-CTCGAAAGAGTCCTGTATTGT-3′). The 18s TET probe was 5′-AGGCAGCAGGCGCGCAAATTAC-3′. Samples were analyzed using the ABI Prism 7700 sequence detection system (Applied Biosystems). Data were analyzed using the relative standard curve method described in the product manual. Additionally, mRNA expression of hamster CD36 (F: 5′-TCAAGGGCATTGGGCAAACAGG-3′ and R: 5′-ATGGCACCGCTCTGCTCAAAC-3′) was determined using Absolute SYBR Green Fluorescein (Thermo Scientific) PCR mix in an iCycler Thermal Cycler instrument (Bio-Rad) with hamster GAPDH as a control (F: 5′-ACGTGTCCGTTGTGGATCTGAC-3′ and R: 5′-CACCACCTTCTTGATGTCCTCATAC-3′). Data were analyzed using the comparative CT method.

## Results

### CHO-GPAT1 cells contained higher intracellular levels of LPA than CHO cells

Rat GPAT1 expression was detected only in the overexpressing cell line (data not shown) with a cycle threshold average of 18.3 (n = 3). Mass spectrophotometry analysis showed that compared with the CHO cells, CHO-GPAT1 cells contained 93% less acyl-CoA, the substrate for GPAT1 (**Table 1**), and 6-times more LPA, the product of GPAT1. DAG in CHO-GPAT1 cells was 41.5% lower than in the CHO cells.

**Table 1:** Overexpression of GPAT1 in CHO cells increased intracellular levels of LPA, and lowered the content of acyl-CoA and DAG.

Lipid intermediates (nmol/g protein)	CHO	CHO-GPAT1
Acyl-CoA	566±317	38 *
Lysophosphatidic acid	236±38	1428±241 *
Diacylglycerol	7608±183	4452±1319 *

Lipid intermediates in CHO and CHO-GPAT1 cells were analyzed by mass spectrometry as described in Methods. Lysophosphatidic acid and diacylglycerol results are expressed as the mean ± SD; n = 3. The acyl-CoA result for CHO-GPAT1 is the average of 2 measurements (32 and 45); * p<0.01.

### Overexpression of GPAT1 in CHO cells increased PPARγ activation

**Figure 2:**
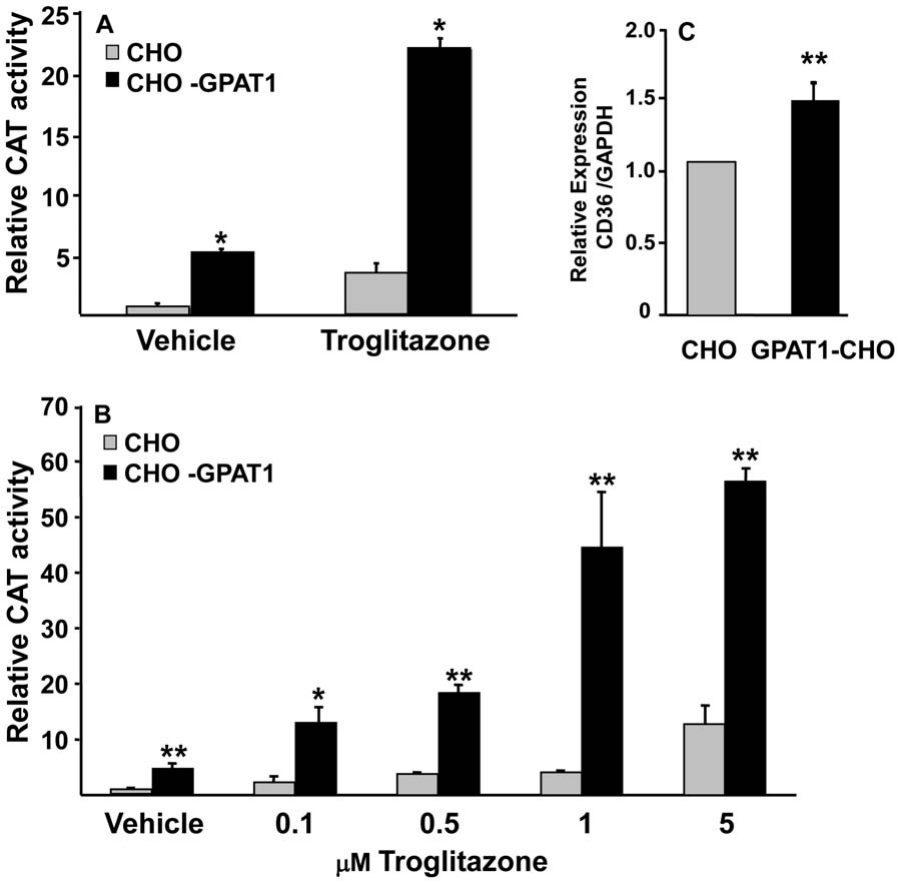
Overexpression of GPAT1 in CHO cells activated PPARγ. (**A**) CHO and CHO-GPAT1 cells were transfected with 0.1 µg of pRLSV40 (internal LUC control) and equal concentrations (0.4 µg) of a PPARγ expression vector, an RXR expression vector, and a PPRE-CAT reporter vector for 24 h, then treated with either 5 µM troglitazone or vehicle (dimethylsulfoxide). (**B**) CHO and CHO-GPAT1 cells were similarly transfected with the described vectors, then treated with either vehicle or troglitazone (0.1 µM to 5 µM). CAT activity 24 h later was normalized to LUC activity (mean +/- SEM; n = 3) and expressed as relative CAT activity. Results are representative of three and two independent experiments, respectively. (**C**) CHO and CHO-GPAT1 cells were grown in MEM as described in the methods. RNA was extracted, and CD36 expression was determined by qRTPCR and normalized to GAPDH expression. Data represent the average of four separate experiments. *p<0.05 and **p≤0.01 when comparing within treatment groups.

To determine whether the increased intracellular content of LPA in the CHO-GPAT1 cells activated PPARγ, CHO cells and CHO-GPAT1 cells were transfected with a PPARγ expression vector and a PPRE-CAT reporter vector. PPARγ activity was 6-fold higher in CHO-GPAT1 cells than in the CHO control cells (**Fig. 2A**). The PPARγ ligand troglitazone increased PPARγ activity 4-fold in CHO cells and an additional 4-fold in the CHO-GPAT1 cells.

In both CHO and CHO-GPAT1 cells, PPARγ activity increased with increasing troglitazone concentrations up to 5 µM (**Fig. 2B**). With all treatments, the CHO-GPAT1 overexpressing cells showed a 4- to 10-fold higher PPARγ activity than their CHO cell counterparts. Because 1 µM troglitazone elicited a near maximal response, it was used for all subsequent experiments. Additionally, mRNA expression of CD36, a PPARγ target, was elevated 2-fold in GPAT1-CHO cells (**Fig. 2C**), suggesting a functional increase in PPARγ activity in GPAT1-overexpressing cells. Studies with a PPARβ/δ expression vector showed very low relative CAT activity in both cell lines, so these isoforms were not investigated further. A PPARα expression vector showed no change in relative CAT activity in the two cell lines, and stimulation with linoleic acid or 1 µM of the PPARα-specific activator Wy14643 did not show a differential effect (data not shown).

### Adding AGPAT2 to CHO-GPAT1 cells decreased PPARγ activity

**Figure 3:**
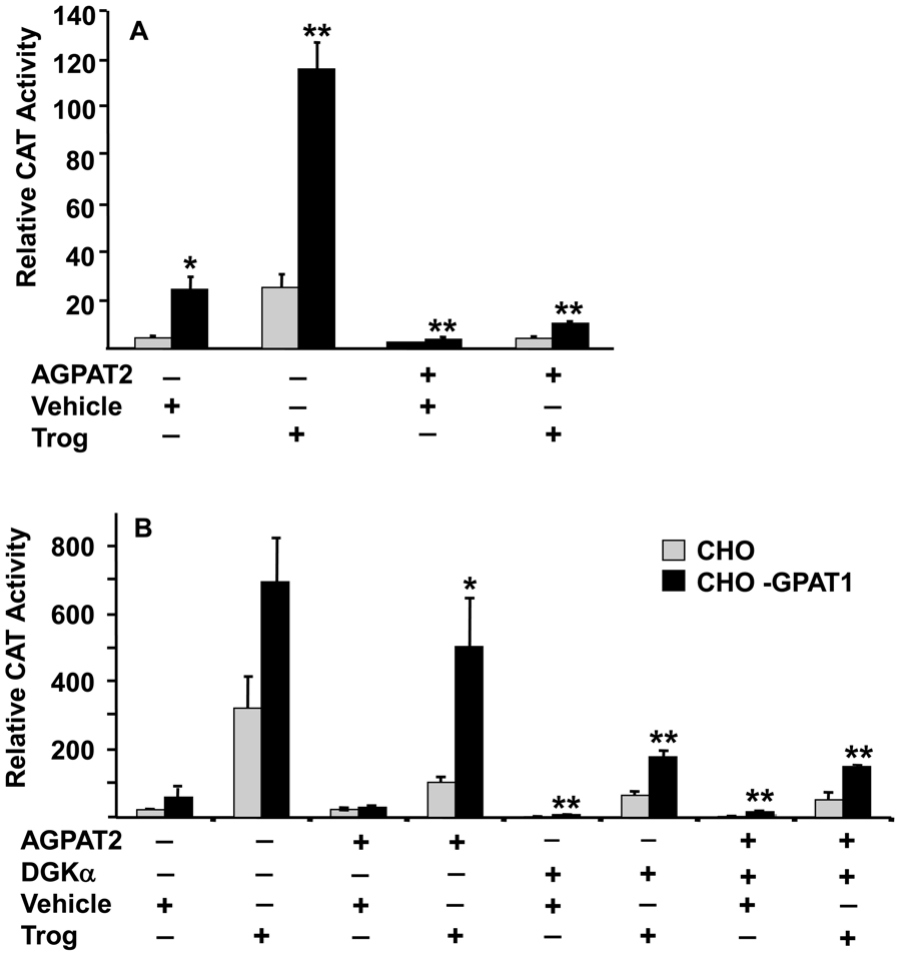
Adding AGPAT2 and DGKα to CHO-GPAT1 cells decreased PPARγ activity. (**A**) CHO and CHO-GPAT1 cells were transfected with 0.1 µg of pRLSV40 (internal LUC control), and equal concentrations (0.4 µg) of expression and reporter vectors, PPARγ RXR, PPRE-CAT, and either empty vector or an AGPAT2 expression vector. (**B**) Cells were similarly transfected with the vectors described plus either a wild-type (inactive) DGKα expression vector (−) or the constitutively active DGKα Δ196 expression vector (+). Twenty-four hours after transfection, cells were treated with either vehicle or 1 µM troglitazone. CAT activity was measured 24 h later and normalized to LUC activity. The results (mean +/− SEM; n = 3) are expressed as relative CAT activity. Results are representative of three and two independent experiments, respectively. *p<0.05 and **p≤0.01 when comparing within treatment groups.

In order to determine whether the increased PPARγ activity in CHO-GPAT1 cells was due to increased intracellular LPA, cells were co-transfected with a 1-acylglycerol-3-phosphate acyltransferase-2 (AGPAT2) expression vector in order to convert LPA to PA and diminish the LPA signal (**Fig. 1**) [16]. Adding the AGPAT2 expression vector markedly lowered PPARγ activity in both CHO control and CHO-GPAT1 cells treated with either the vehicle or troglitazone (**Fig. 3A**). The AGPAT2-mediated decrease in PPARγ activity was consistent, although the extent of the decrease varied due to variation of luciferase sensitivity between experiments (compare **Fig. 3A and 3B**).

### Converting DAG to PA decreased PPARγ activity

Because DGKα catalyzes the phosphorylation of DAG to form PA, the transfection of constitutively active diacylglycerol kinase α (DGKα) either alone or together with AGPAT2, allowed us to analyze the effects of increasing cellular PA and of decreasing both LPA and DAG (**Fig. 1**). Co-transfecting AGPAT2 plus constitutively active DGKα lowered PPARγ activation similar to AGPAT2 alone and decreased the effect of troglitazone in both cell lines (**Fig. 3B**). CHO-GPAT1 cells transfected with DGKα alone showed a PPARγ response similar to cells co-transfected with both AGPAT2 and DGKα, a result which is consistent with inhibition of PPARγ by PA. Of interest is the recent report that cyclic phosphatidic acid is an inhibitor of PPARγ [22]. Regardless of treatment, PPARγ activity remained higher in CHO-GPAT1 cells than in CHO cells.

### LPA and PA displace [^3^H]rosiglitazone

Radioactive competitive binding assays confirmed that LPA was a PPARγ ligand. Palmitoyl-LPA, oleoyl-LPA, 1-palmitoyl, 2-oleoyl-PA, and 1,2 dioleoyl-PA competitively displaced between 20–30% of [^3^H]rosiglitazone with micromolar affinity from the PPARγ ligand binding domain (**Table 2**), as described previously [4], [5]. PA appeared to be more potent than LPA, but LPA binding was similar to that described in previous reports [4], [5].

**Table 2:** LPA and PA displace [^3^H]rosiglitazone from PPARγ-LBD.

Glycerolipid species	Percent displacement of [^3^H]rosiglitazone
*sn*-1-18∶0-LPA	25.9±1.5
*sn*-1-16∶0-LPA	29.6±1.2
*sn*-1, 2-di18∶1-PA	24.2±0.8
*sn*-1-16∶0, 2-18∶1-PA	21.7±0.9

Increasing concentrations of lipids were evaluated for the ability to displace [^3^H]rosiglitazone from PPARγ LBD. LPA, lysophosphatidic acid; PA, phosphatidic acid. The results are expressed as the mean ± SEM; n = 4 at 100 µM.

### Extracellular addition of LPA, PA, or diC8∶0 did not increase PPARγ activity

**Figure 4:**
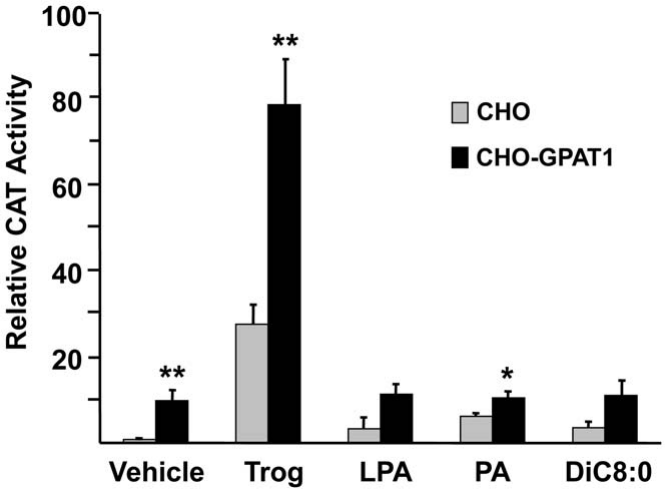
Treating CHO and CHO-GPAT1 cells with LPA, PA, or DiC8∶0 did not enhance PPARγ activity. CHO and CHO-GPAT1 cells were transfected with 0.1 μg of pRLSV40 (internal LUC control), and equal concentrations (0.4 μg) of a PPARγ expression vector, an RXR expression vector, and a PPRE-CAT reporter vector. Twenty-four hours later, the transfected cells were treated with vehicle (0.1% BSA), 1 μM troglitazone, 5 µM oleoyl-LPA, 5 µM dioleoyl-PA, or 10 µM DiC8∶0. CAT activity was measured 24 h later and normalized to LUC activity. The results (mean +/− SEM; n = 3) are expressed as relative CAT activity. Results are representative of two independent experiments. *p<0.05 and **p≤0.01 when comparing within treatment groups.

LPA is formed from membrane phospholipids by phospholipases D and A2 and regulates internal signaling pathways via G protein-coupled cell surface receptors [3], [23], [24]. LPA entry into cells is problematic. Entry is inhibited by albumin [6] and by cell-specific enzymes like ecto-lipid phosphate phosphohydrolase (LPP) [25]. Exogenously provided LPA can activate a G protein-coupled signaling pathway that may affect PPARγ activity [3]. For example, extracellular LPA inhibits adipogenesis via the LPA_1_ receptor [26], and the LPA_1_ receptor is expressed by CHO cells [24]. In order to make certain that the effects on PPARγ were not due to effects of external LPA or PA on G protein-coupled receptors and to learn whether DAG might be playing a role in the observed effects, we added LPA, PA, or dioctanoyl glycerol to the culture medium (**Fig. 4**). Dioctanoylglycerol is a cell permeable DAG [27], and intact PA does not enter cells [28]. Compared with the vehicle control, these additions had no effect on PPARγ activity. Thus, neither DAG, LPA nor PA added externally were able to increase PPARγ activity.

### Adding fatty acids to CHO-GPAT1 cells increased PPARγ activity

**Figure 5:**
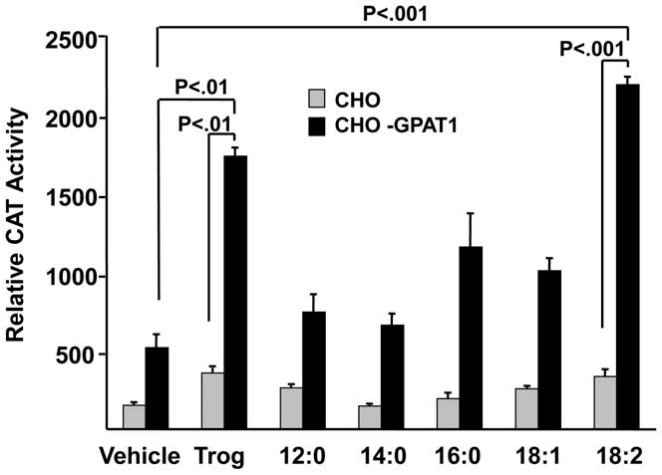
Overexpression of GPAT1 in CHO cells enhanced the effects of fatty acid treatments on PPARγ activity. CHO and CHO-GPAT1 cells were transfected with 0.1 µg of pRLSV40 (internal LUC control), and equal concentrations (0.4 µg) of a PPARγ expression vector, an RXR expression vector, and a PPRE-CAT reporter vector, as described. Twenty-four hours later, transfected cells were treated with either vehicle (0.1% BSA), 1 µM troglitazone, or 250 µM fatty acid (lauric acid (12∶0), myristic acid (14∶0), palmitic acid (16∶0), oleic acid (18∶1), or linoleic acid (18∶2)). CAT activity was measured 24 h later and normalized to LUC activity. The results (mean +/− SEM; n = 3) are expressed as relative CAT activity and are representative of two independent experiments. Results were analyzed by ANOVA and individual comparisons by Fischers LSD test for multiple comparisons.

Because the effects of GPAT1 and troglitazone were synergistic, we wondered whether PPARγ ligands like polyunsaturated fatty acids might act similarly. PPARγ activity is strongly activated by 12∶0 and 18∶2 and weakly activated by 18∶1 and 16∶0 [13]. In CHO cells, both troglitazone and 18∶2 increased PPARγ activity 2-fold compared to the vehicle control, but other fatty acids were less effective (**Fig. 5**). In the CHO-GPAT1 cells, 18∶2 and troglitazone treatments were also equivalent; each increased PPARγ activity 3- to 4-fold compared to the vehicle. None of the other fatty acids had an effect greater than treatment with the vehicle alone.

## Discussion

LPA, an intermediate in the glycerol-3-phosphate pathway of glycerolipid synthesis had previously been identified as a PPARγ ligand and agonist [4], [5], but when added exogenously, it required tridentate sulfonamid or dimethyl sulfoxide to enhance its entry into RAW264.7 or CV-1 cells [4], [6]. Evidence for functional activation of PPARγ by exogenously added LPA was inferred by an increase in the PPARγ target CD36 on the surface of primary human monocytes [4]. However, because LPA does not readily enter cells, the physiological significance of these observations has remained uncertain.

Our study confirmed previous reports that showed that LPA can partially displace [^3^H]rosiglitazone [4], [5] and suggested the possibility that the PPARγ binding site might simultaneously accommodate both thiazolidinedione and a second ligand. Dual binding provides a possible explanation for the synergistic effect of troglitazone and LPA as well as for the apparent inhibitory effect of PA on PPARγ-mediated expression of CAT activity. We attribute the increase in PPARγ activity to the increased LPA levels since it is known that LPA can compete with the selective PPARγ ligand rosiglitazone for binding to PPARγ [4], and as little as 1 nM of the ether analog AGP is able to displace ∼45% of bound [^3^H]rosiglitazone [5]. However, our results also showed that the overexpression of GPAT1 in CHO cells enhanced the effect of troglitazone on PPARγ activity, suggesting that troglitazone and LPA worked synergistically to enhance PPARγ activity. Synergistic enhancement is consistent with the suggestion that rosiglitazone and AGP bind tightly to PPARγ and that their binding sites, although overlapping, are not in the same position, since the two ligands cannot fully displace each other [5]. A less likely possibility is that LPA might act indirectly by modifying the function of a PPARγ coactivator.

Despite studies that show direct LPA binding to PPARγ, LPA molecules that are produced within cells via a pathway of *de novo* synthesis have not previously been considered to function as PPARγ agonists [29]. In order to investigate this possibility, we used a CHO cell line that stably overexpresses GPAT1 [12]. These cells have a 3.8-fold increase in GPAT1 activity and contain a higher triacylglycerol mass than control cells, suggesting that the intermediates of glycerolipid synthesis, including LPA, might be elevated. This hypothesis was confirmed by a mass spectrophotometric analysis that showed a 6-fold increase in LPA levels in the CHO-GPAT1 cells and acyl-CoA levels 93% lower than those in control CHO cells. These results are opposite those observed in *Gpat1^−/−^* mice in which the liver LPA content is 50% lower and acyl-CoA content is 3-fold higher than in wild type controls [10]. Because CHO-GPAT1 cells have higher levels of LPA and TAG, we also expected to find increased DAG content, but DAG was 50% lower in the overexpressing cell line, perhaps because it was rapidly converted to TAG [12].

Decreased PPARγ activity has also been observed in another study in which cells were transiently transfected with an AGPAT2 expression vector and then treated with extracellular LPA; in that study, however, AGPAT expression did not inhibit PPARγ in cells treated with rosiglitazone or two other PPARγ agonists [4]. In contrast, our study showed that the AGPAT2 expression vector markedly lowered PPARγ activity even in the presence of troglitazone, suggesting the possibility that PA, endogenously synthesized by AGPAT, may inhibit PPARγ. On the other hand, PA may have had an inhibitory effect in our study, only because we used troglitazone, a relatively weak PPARγ ligand.

LPA can regulate a variety of signaling pathways within cells by binding to G protein-coupled receptors located on the cell surface [30], and it was possible that the effects on PPARγ might be mediated by this type of pathway if the LPA synthesized by GPAT1 could cross the plasma membrane to leave the cell and then interact with LPA1 receptors present on the plasma membrane [24]. In order to ascertain whether the increased PPARγ activity we observed in CHO-GPAT1 cells was due to intracellular versus secreted LPA, we added LPA to the medium. Because externally added LPA had no effect on the PPARγ reporter, endogenous and intracellular LPA must have activated PPARγ.

In order to increase the LPA pool available to activate PPARγ, CHO and CHO-GPAT1 cells were transfected with the PPARγ expression vector and PPRE-CAT reporter and then treated with different fatty acids at 250 µM. Although 18∶2 strikingly increased PPARγ activity in the CHO-GPAT1 cells, the observed increase in PPARγ activity in this and other studies [13] might be due either to direct binding of the fatty acid to PPARγ or to an increase in the total amount of LPA available as an agonist.

The major finding of this study is that LPA acts as a PPARγ agonist when it is formed intracellularly as an intermediate in the glycerolipid biosynthetic pathway. This finding is important in light of the current focus on the association of obesity and hepatic steatosis, because stimulation of PPARγ by thiazolidinedione drugs like troglitazone alleviates insulin resistance [31]. In hepatic steatosis, both PPARγ and GPAT1 are normally upregulated [32], [33], and adenovirus-mediated overexpression of GPAT1 in rat liver promotes the development of hepatic steatosis within 5–7 days [11]. Upregulation of GPAT1 increases its LPA product [11], which could physiologically synergize with other PPARγ ligands to enhance hepatic steatosis. In a related study in which *Gpat1^−/−^* mice were crossed with *ob/ob* mice, hepatic TAG content was reduced by 59% [34], suggesting that GPAT1 is responsible for most of SREBP-1 regulated hepatic TAG accumulation. In the current study, upregulation of PPARγ is supported by an increase in the PPARγ target CD36. Although our study used cells that stably over-express GPAT1, GPAT specific activity in the CHO-GPAT1 cells is considerably lower than that normally measured in liver and adipocytes. Thus, the enhanced activity of GPAT that activated PPARγ activity in this study is relevant to GPAT specific activities that are normally present in the major lipogenic tissues.

Our study showing synergism between LPA and troglitazone also suggests that different ligands activating a common nuclear receptor may induce different effects, including different sets of genes and biological activities [1]. Future experiments to test the physiological effects of LPA generated by GPAT will require the use of mammalian cells that have both a high expression of PPARγ and a low activity of GPAT that can be enhanced by transfection.
